# Contribution of the base of the heart to MR measures of left ventricular mass, volumes and scar in patients with myocardial infarction

**DOI:** 10.1186/1532-429X-11-S1-P53

**Published:** 2009-01-28

**Authors:** Carissa G Fonseca, Tariq Balawi, James Sayre, Paul Finn

**Affiliations:** UCLA, Los Angeles, CA USA

**Keywords:** Left Ventricular Mass, Delay Enhancement, Infarct Border, Delay Enhancement Image, Cardiac Cine

## Introduction

In planimetric measurement of left ventricular (LV) mass and volumes, selection of the most basal short-axis cine MR image plane is generally not standardized and may affect calculation of relative infarct size in post-myocardial infarct (MI) patients.

## Purpose

To study the effect of LV base-plane selection on MR measures of LV mass, volumes, ejection fraction (EF) and relative infarct size in post-MI patients.

## Methods

SSFP cardiac cine and multi-shot inversion recovery spoiled gradient echo delayed enhancement (DE) images were acquired in 14 patients with documented MI (3 F; mean age 59.6 ± 10.4 years), at 1.5 T. Cine and DE images were acquired in corresponding short-axis planes (voxel size ≤ 2.5 × 2.0 × 10.0 mm; slice gap ≤ 2 mm) from LV base (mitral valve plane) to apex. An expert observer used Argus software (Siemens Medical Solutions) to manually outline LV epi- and endomyocardial contours on cine images at end-diastole (ED) and end-systole (ES) using each of 3 methods for choosing the most basal LV slice: #1 base-plane showing 100% of myocardium at ED and corresponding ES plane, #2 base-plane selected separately for ED and ES, with 100% of myocardium visible at both phases, #3 base-plane showing 100% of myocardium at ES and corresponding ED plane. LV mass was averaged over ED and ES for each method. EF, and ED and ES-volumes were also calculated. Infarct borders were manually outlined on DE images. Infarct size (Infarct%) was expressed as a percentage of LV mass measured on the cines (in each method). Comparisons were made using repeated measures ANOVA, with Bonferroni-corrected post-hoc P-values.

## Results

LV mass was largest in method #1 and smallest in #3 (138.5 ± 31.2 vs. 133.0 ± 29.3 vs. 124.8 ± 27.3 g, P < 0.01 for all comparisons). Method #2 produced a larger EF (51.3 ± 8.9%) vs. #1 (43.8 ± 9.4%) or #3 (43.9 ± 10.8%); P < 0.01. Infarct% was smallest in method #1 and differed significantly between all methods (9.7 ± 4.9 vs. 10.2 ± 5.3 vs. 10.9 ± 6.0%, P < 0.05). ED volume was lower in #3 than in #1 or #2 (P < 0.01) and ES volume was higher in #1 than in #2 or #3 (P < 0.01).

## Conclusion

Selecting the base-plane based on the anatomy visible in only one phase, will lead to miscalculation of MRI measures of LV mass, volumes and relative infarct size, due to through-plane motion of the heart over the cycle. To standardize measurements and to ensure that the same tissue is being measured at both phases, the most basal slice should be selected independently for ED and ES based on the visible anatomy.

